# Design and operation of a low-cost and compact autonomous buoy system for use in coastal aquaculture and water quality monitoring

**DOI:** 10.1016/j.aquaeng.2017.12.002

**Published:** 2018-01

**Authors:** Wiebke Schmidt, David Raymond, David Parish, Ian G.C. Ashton, Peter I. Miller, Carlos J.A. Campos, Jamie D. Shutler

**Affiliations:** a Centre for Geography, Environment and Society, University of Exeter, Penryn Campus, Penryn TR10 9FE, United Kingdom; b Offshore Renewable Energy Group, College of Engineering, Mathematics and Physical Sciences, University of Exeter, Penryn Campus, Penryn TR10 9FE, United Kingdom; c Plymouth Marine Laboratory, Prospect Place, The Hoe, Plymouth PL1 3DH, United Kingdom; d Centre for Environment, Fisheries & Aquaculture Science (Cefas), Weymouth Laboratory, Barrack Road, The Nothe, Weymouth DT4 8UB, United Kingdom

**Keywords:** Buoy sensors, Robust, Water quality, Aquaculture, Shellfish

## Abstract

The need to ensure future food security and issues of varying estuarine water quality is driving the expansion of aquaculture into near-shore coastal waters. It is prudent to fully evaluate new or proposed aquaculture sites, prior to any substantial financial investment in infrastructure and staffing. Measurements of water temperature, salinity and dissolved oxygen can be used to gain insight into the physical, chemical and biological water quality conditions within a farm site, towards identifying its suitability for farming, both for the stock species of interest and for assessing the potential risk from harmful or toxic algae. The latter can cause closure of shellfish harvesting. Unfortunately, commercial scientific monitoring systems can be cost prohibitive for small organisations and companies to purchase and operate. Here we describe the design, construction and deployment of a low cost (<£ 5000) monitoring buoy suitable for use within a near-shore aquaculture farm or bathing waters. The mooring includes a suite of sensors designed for supporting and understanding variations in near-shore physical, chemical and biological water quality. The system has been designed so that it can be operated and maintained by non-scientific staff, whilst still providing good quality scientific data. Data collected from two deployments totalling 14 months, one in a coastal bay location, another in an estuary, have illustrated the robust design and provided insight into the suitability of these sites for aquaculture and the potential occurrence of a toxin causing algae (*Dinophysis* spp.). The instruments maintained good accuracy during the deployments when compared to independent in situ measurements (e.g. RMSE 0.13–0.16 °C, bias 0.03–0.08 °C) enabling stratification and biological features to be identified, along with confirming that the waters were suitable for mussel (*Mytilus* spp.) and lobster (*Homarus gammarus*) aquaculture, whilst sites showed conditions agreeable for *Dinophysis* spp.

## 1 Introduction

Near-shore coastal waters are highly heterogeneous in both space and time, are regions of high biological activity and can be subject to dramatic and rapid changes due to strong winds and tidal currents (Smyth et al., 2010). For an expanding populations, shellfish farming has the potential to supply high quality protein based products in cost-effective and sustainable farming systems. A viable shellfish industry depends on productive waters that are free from pollution. However, episodes of poor water quality and coexistence with other water-based activities have motivated many farmers to invest in offshore farming operations (FAO, 2016, Wright, 2016, Science for Environment Policy, 2015). When identifying new aquaculture locations, such as for bivalve molluscs, it is prudent to first characterise the water quality. Similarly, such information and monitoring is also likely to be valuable once a farm is operational. Standards for European water quality for shellfish waters have been established under the European Union Water Framework Directive (EU, 2000). Failure to comply with these standards can have negative impacts on aquaculture businesses, including financial loss and lower customer confidence, due to harvesting closures or downgrades in shellfish water classification. Typically, EU and international monitoring programmes rely on in situ sampling to assess water quality. However, these environmental data are temporally sparse and often irregularly sampled (e.g. fortnightly or monthly sampling governed by weather conditions), and are unlikely to fully characterise the temporal variations in near-shore coastal waters. Deploying water quality monitoring instruments (e.g. on a buoy or mooring) can allow the high frequency collection of physical, chemical and biological properties of the water column within a farm, allowing the temporal variations in water quality to be characterised and the drivers of these changes in the water quality to be better understood. For example, information on biological production (via dissolved oxygen measurements) and water column stratification (via temperature and salinity measurements) can be easily collected. However, the deployment and operation of permanent scientific monitoring buoys, as used by national and international agencies and harbour authorities, are typically expensive (e.g. capital cost of >£ 0.5–1 million) and thus few of them exist. They provide excellent temporal coverage, but sparse spatial coverage in the heterogeneous coastal zones and this approach is cost prohibitive for small to medium sized businesses to purchase and operate (Smyth et al., 2010, Brewin et al., 2015, Defeo et al., 2009, Glasgow et al., 2004). Whereas clearly such monitoring could provide a rich source of information for shellfish farm management.

Here we describe the development, integration, deployment and initial operation of a low-cost (total cost <£ 5000) autonomous buoy system. The buoy system is generic in design and integrates sensors for monitoring various water quality parameters, such as seawater temperature, salinity, water level and dissolved oxygen. We report on the application of the system to monitor the biological and physical oceanography in two coastal sites in the North East Atlantic. The first test site, a coastal bay (St Austell Bay) contains two areas leased for mussel aquaculture (*Mytilus* spp.) which is currently being assessed for its potential for lobster aquaculture (*Homarus gammarus*). Additionally, seven recognised bathing water beaches are found within the bay. The second test site is within a large estuary (Fal estuary) which is one of three oysters fisheries in England and Wales, harvesting native oysters (Long et al., 2017). These coastal waters along the south coast of the United Kingdom are predominately exposed to south-westerly weather systems. Storm force winds occur regularly, whilst exposure to the Atlantic Ocean leads to potentially destructive waves, particularly during winter months (Smyth et al., 2010, Smith et al., 2012). These conditions can present a challenge for long-term deployment of moorings and scientific instruments. Therefore, the mooring itself needs to be robust to hold the instrument buoy in position even when exposed to strong winds and large waves and so a simple mooring solution was also evaluated.

## 2 Development of the buoy system

### 2.1 Integration of the buoy system

To allow the continuous collection of physical and biochemical parameters the sensors used here were chosen to: minimise power requirements and consumption, size, weight, cost and complexity of installation and maintenance (in capital cost and staffing time), whilst maximising measurement accuracy, precision, sampling frequency and ease of use. These criteria cover and address both scientific and aquaculture business requirements and constraints.

**Fig. 1:**
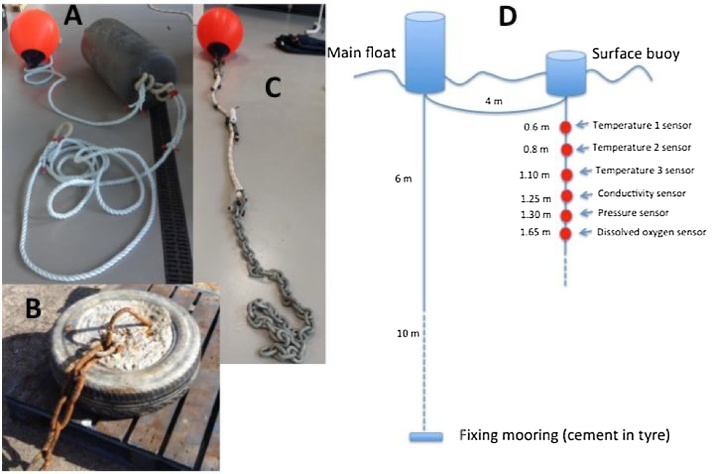
The buoy system showing (A) the connection to 100 L float, (B) the tyre filled with concrete, (C) the instrument string and (D) the schematic of the complete mooring with the positions of the scientific instruments labelled along the instrument string. Solid line = fibre rope, dotted line = steel chain used in mooring line which also acts as a weight to keep the instrument string vertical in the water column.

In collaboration with the local shellfish farmer the mooring configuration was designed following the design rules of existing and reliable farm moorings. The mooring is comprised of two parts (Fig. 1a–d). The first part consists of a 100 L float attached to a 6 m long rope (type: 3 strand laid polypropylene; diameter 22 mm), leading to a 10 m long galvanised steel chain (gauge: 16 mm) and a concrete filled car tyre, which acts as an anchor to the seabed. A second surface buoy (type A3) is connected to the main 100 L float via a 4 m long rope (type: 3 strand laid polypropylene; diameter 22 mm). Hanging vertically from this A3 buoy is a 3 m rope (type: double braided polyester; diameter 22 mm) holding the sensors with a weighting chain (length: 2 m; weight: approx. 15 kg) attached at the bottom.

This two-piece configuration meant that the main mooring could be placed in advance of the connection of the instrument chain. Similarly, the lightweight instrument chain can be easily lifted using a boat hook and a low power winch, such as that used by shellfish farmers to lift strings of mussels. Furthermore, during maintenance and offloading of data the main mooring does not need to be lifted and is robust enough to moor the vessel. The scientific instruments attached to the A3 buoy can move freely (circulate) around the main 100 L float (e.g. as the mooring is influenced by tidal currents). This has the potential to compromise the vertical orientation of the instrumentation string. In order to account for this movement, a pressure (level) sensor was included at 1.30 m depth (Fig. 1d) as this allows the actual depth and vertical orientation of the instruments to be known. Each of the instruments themselves (or their protective housing) and a plastic identification tag with contact telephone numbers were directly stitched onto the instrument string rope.

### 2.2 Instrumentation of the buoy system

To enable the monitoring of physical and biochemical parameters for understanding the drivers of changes in water quality, temperature, salinity and dissolved oxygen (Fig. 1d) were chosen as the initial parameters to monitor (EU, 2000). Interest in the formation and changes in stratification (as an indicator of changes in biological activity) meant the desire to measure temperature a multiple depths.

Following the requirements described in Section 2.1 the HOBO U-series sensors were chosen: three temperature sensors (for different depths), a conductivity and salinity sensor, a pressure sensor and a dissolved oxygen (DO) sensor (Fig. 1d). Each sensor attached to the instrument string (Fig. 1d), includes an internal data logger and lithium batteries. Furthermore, each sensor monitors its battery voltage and logs a ‘bad battery’ event should it fall below 3.2 V. The common design of these sensors means that all data can be electronically offloaded using a single hand-held and waterproof data shuttle that requires no computer or cables for its operation. The sensors, except the DO sensor, are factory calibrated and so upon delivery they were ready to deploy. An overview of each sensor’s characteristics including size, weight, measurement ranges, manufacturers’ accuracy and drift are described in Table 1.

**Table 1:** Specifications of deployed sensors (Source: manuals of loggers provided by HOBO).

Parameter	Temperature	Conductivity	Dissolved oxygen	Pressure
Model	Hobo**^®^** Tidbit v2 Logger	Hobo**^®^** Conductivity Logger	Hobo**^®^** Dissolved Oxygen Logger	Hobo**^®^** Water level pressure
Size & weight	30 × 41 × 17 mm; 23 g	31.8 mm diameter × 165 mm; 6.3 mm mounting hole; weight 193 g	39.6 mm diameter × 267 mm; weight 464 g	31.8 mm diameter × 152.4 mm length; weight 154 g
Measurement range	−20 to +30 °C	1,000 to 55,000 μS cm^−1^	0 to 30 mg L^−1^	0 to 207 kPa
Accuracy	±0.2 °C over range from 0 to 50 °C	±5% of reading, in waters within a range of 3,000 μS cm^−1^, waters with greater variation can have greater error.	±0.2 mg L^−1^ up to 8 mg L^−1^ & ±0.5 mg L^−1^ for 8–20 mg L^−1^	±0.3%
Drift	0.1 °C per year	Up to 12% sensor drift per month, exclusive of drift from fouling.	Not stated	Not stated
Calibration	Factory calibrated	Factory calibrated, however monthly start & end-point calibration is recommended to compensate for drift	3-step calibration prior sensor cap initialisation	Factory calibrated
Logging rate	Set to log every 10 min (range from sec – 18 h)			
Memory	Approx. 42,000 temperature measurements	18,500 temperature and conductivity measurements	21,700 sets of DO & temperature measurements	Approx. 21,700 pressure & temperature samples
Deployment depth	Max 300 m	Max 70 m	Max 100 m	Max 9.14 m
Battery	Internal battery with typical life of 5 years; non-replaceable	3.6 V lithium battery; typical life of 3 years	3.6 V lithium battery; typical life of 3 years; factory replaceable	2/3 AA, 3.6 V lithium; factory-replaceable
Accessories		Housing for protection	Anti-Fouling guard	–

For the calibration of the DO sensor (and as per the manufacturer guidelines) a 3-step calibration was followed. The sensor was first calibrated to 100% saturation by placing it in water-saturated air. The sensor was then covered with the calibration boot (as supplied) with a sponge wetted with fresh tap water for approximately 15 min to allow the sensor to reach temperature equilibrium. Afterwards the sensor was placed in a 0% saturated oxygen environment using a 2 M sodium sulphite solution (Onset, U-26 calibration solution).

The logging interval for each sensor was set to 10 min and all internal clocks were set using a common reference. This sampling period meant that each sensor was capable of operating continuously for up to twelve months. The shuttle is compatible with all HOBO U-series sensors and has a data capacity of 63 logger readouts of up to 64 Kilobytes (kB) each. One transfer of the full 64 kB (logger–to-shuttle transfer) takes about 30 s and the shuttle operates with two 1.5-V AA batteries. After offloading the data, sensors can be cleaned of any biofouling using a toothbrush and fresh water. In order to minimise potential biofouling, the DO and conductivity sensor were both placed in the manufacturer provided antifouling guards and housing.

## 3 Buoy deployment and operation

### 3.1 Deployment: St Austell Bay and Fal estuary

From 7 October 2015–9 August 2016, the buoy system was deployed close to a shellfish farm in St. Austell bay, Cornwall, United Kingdom (50° 18.92′ N 004° 43.70′ W) and all parameters were measured every 10 min for nine months. Monthly, data were offloaded using the data shuttle. The readout of one month of logged data comprised between 15 and 20 kB and after the data transfer all sensors were cleaned of any biofouling, which occurs as a natural process as soon as a substratum is deployed within the marine environment (Lehaitre et al., 2008). Independent temperature and salinity measurements were collected during each maintenance visit, but this independent instrument was later found to be faulty and so the measurements were deemed unsuable for evaluating any drift in the sensors due to biofouling. Therefore to further test the performance of the complete buoy system and of any degradation and impact of biofouling on the sensors, the system was deployed for a second period in the Fal estuary for five months (21 November 2016 to 9 May 2017; Turnaware Bar, 50° 12.349′ N 005° 2.015833′ W). During this second deployment independent conductivity, temperature and depth (CTD) profiles were taken during nine maintenance visits to the buoy using a calibrated handheld CastAway**^®^** CTD.

### 3.2 Data processing and quality control

After offloading all sensor measurements, all data were exported to comma separated variable files using the HOBO software (version 3.7.8). Conductivity readings were converted to salinity (in PSU) using the ‘convert_RtoS’ from the R package ‘marelac’, using conductivity ratio (conductivity observations in S m^−1^ divided by a standard conductivity of 4.2914 S m^−1^), pressure (in bar) and water temperature data from the internal temperature sensor housed in the conductivity sensor (T **°**C) (Soetaert et al., 2015). To calculate the alignment of the buoy within the water column, the water level was determined from the pressure sensor data using the HOBO software.

After manual inspection of the data and guided by the U.S. Integrated Ocean Observing System (U.S. Integrated Ocean Observing System, 2015) the following quality control rules were followed:

1. Observations outside of the temperature (−2 to 30 °C), salinity (1000 and 55,000 μS cm^−1^) and dissolved oxygen (0–30 mg L^−1^) ranges were discarded (ranges were based on Table 1 and the freezing point of seawater at 35 PSU at the waters surface).
2. The first two hours of measurements from all sensors were discarded (e.g. after initial deployment and each cleaning) to allow the sensors to (re)stabilise.
3. Single anomalous values were discarded, where anomalous is defined as abrupt changes between consecutive measurements.
4. If a gradual roll off in measurements was observed in the second half of the deployment period, (i.e. prior to cleaning), then the peak measurement was retained and all measurements thereafter were discarded until the end of the deployment period (as this was assumed to be degradation of measurement quality due to biofouling).
5. All measurements from all sensors were discarded when the pressure sensor indicated that the instrument string was at the surface (i.e. floating and tangled).

## 4 Results and discussion

### 4.1 Comparison of sensor data with independent measurements

The two deployments resulted in a total of 37,828 (St Austell Bay) and 24,305 (Fal estuary) measurements. 0.015% of the St Austell Bay and none of the Fal estuary temperature measurements failed criteria number one. Comparing the mean buoy sea temperature obseravations in St Austell bay (at 1.1 m) with the nearest Channel Coastal Obsveratory buoy (Looe Bay, 50° 20.33′N, 04° 24.64′W) showed that the observations from the buoy instruments over the complete deploymet were within the range measured by the Looe Buoy (mean sea temperature at Looe from October 2015 to July 2016 = 12.2 **°**C ± 2.1 standard deviation and mean sea temperature from the buoy temperature sensor at 1.1 m = 12.4 **°**C ± 2.0 standard deviation). As expected for near-shore coastal waters, the salinity observations varied considerably over the course of deployment. However, from initial assessment of the data and the obsverations during the maintencance visits, it became clear that biofouling was influencing the salininty measurements. Biofouling is known to act as a primary limiting factor in terms of measurement accuracies and deployment longevity (Manov et al., 2004). The complex and highly biolologically active nature of coastal waters means there can be considerable spatial and temporal variation in biofouling. Criteria three and four addressed this issue and resulted in the removal of 19.71% of the St Austell Bay and 0.02% of the Fal estuary conductivity measurements. Over the nine month deployment in St Austell bay, DO observations ranged from 2.99 to 15.76 mg L^−1^ and 1.31% of DO measurements (n = 37,828) were removed as a result of the quality control criteria. DO observations for the Fal estuary ranged from 7.08 to 15.03 mg L^−1^ and 0.02% of DO measurements were removed by following the quality criteria.

During the maintenance trips in the Fal estuary, independent measurements were used to assess the differences between sensor measurements and those collected by the CTD. The root mean square error (RMSE) and bias between these two sets of measurements (assuming the CTD measurements to be truth) were calculated and these are listed Table 2.

**Table 2:** Comparison between CTD measurements and Hobo sensors (n = 9 observations).

Endpoint	Instruments	Deployed depth	RMSE	Bias
Temperature (°C)	CTD vs. Tidbit 1	0.45/0.3 m	0.16 °C	0.08 °C
	CTD vs. Tidbit 2	0.45/0.4 m	0.12 °C	0.03 °C
	CTD vs. Tidbit 3	0.75/0.75 m	0.13 °C	0.03 °C
	CTD vs. U24 Cond. Logger	1.04/1.1 m	0.2 °C	0.19 °C
Conductivity (μS/cm)	CTD vs. U24 Cond. Logger		1204.1 μS cm^−1^	−405.81 μS cm^−1^
Salinity (PSU)	CTD vs. U24 Cond. Logger		1.3 PSU	−0.53 PSU

Differences between the buoy temperature sensor measurements and CTD measurements were low (RMSE = 0.12 °C–0.16 °C, bias of 0.03–0.08), are consistent with previous laboratory assessments of these sensors (Brewin et al., 2015) and are lower (higher accuracy and precision) than the manufacturer specifications (Table 1). The temperature readings from the conductivity sensor (required for the salinity calibration) showed the highest RMSE of 0.2 °C and bias of 0.19 °C, but again these are within the range provided by the sensor manufactures (Table 1). The bias of 0.19 °C illustrates the warming that occurs within the conductivity sensor housing and illsutrates why this measurements is necessary for the conductivity to salinity conversion. Additionally, the RMSE for the conductivity measurements was low (RMSE = 1204.1 μS cm^−1^) and within the manufacturers stated accuracy of the 5% of the sensor range (Table 1).

These results show good agreement between the quality controlled sensor data and the independent CTD measurements and illustrate the stability of the sensors.

### 4.2 Mooring performance and storm events, St Austell Bay

The buoy system successfully delivered on all aspects of its design. The sensors were self-sufficient in power and continuously recorded the environmental parameters without any failure. During each maintenance trip it was easy and quick to offload the data and clean the instruments. It took around 20 min in total to anchor onto the main mooring, lift the instrument string (A3 buoy), offload and clean the instruments and return the instruments back to the water.

**Fig. 2:**
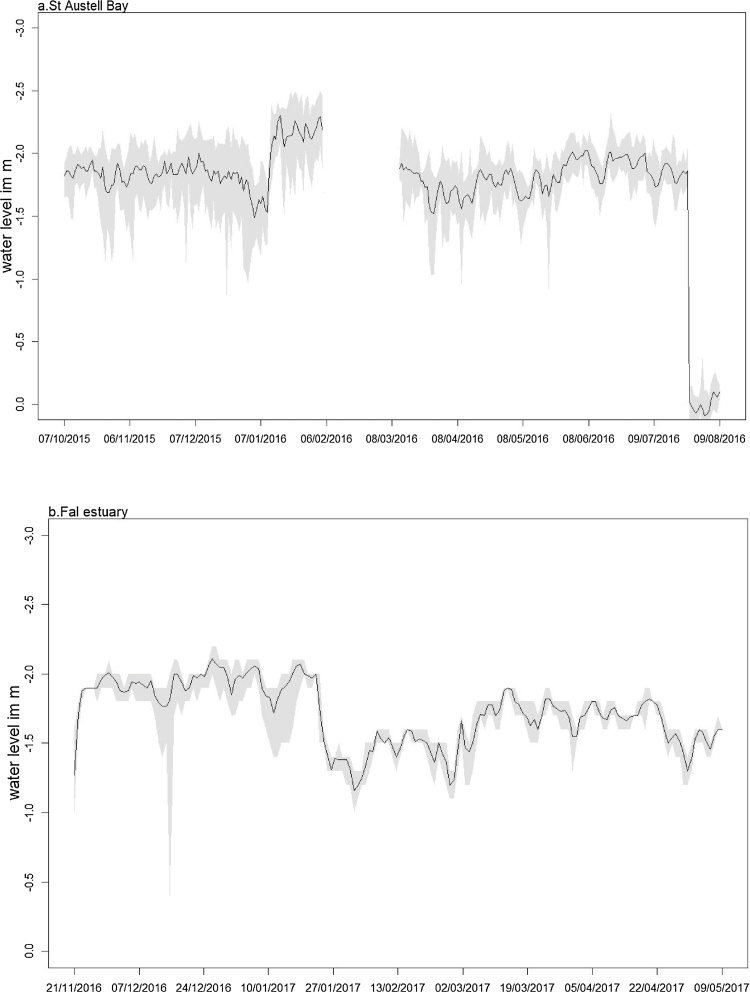
(a) Mean water level (in m, black line with the minimum and maximum water level represented as shaded area) at 1.30 m depth for St. Austell Bay from 7 October 2015–9 August 2016 and (b) for Fal estuary from 21 November 2016 to 9 May 2017.

The buoy instrument string system maintained its vertical orientation for the majority of the 14-month deployment (Fig. 2). It did become tangled around the main 100 L float at month nine of the St Austell Bay deployment (indicated by the instrument depth becoming ∼0.5 m). This entanglement occurred during a period of storms where the observed wind speed ranged from 0 to 13.5 m s^−1^ and wave heights were between 0.19 and 1.9 m (Channel Coastal Observatory buoy, Looe bay (Channel Coastal Observatory, 2015)).

During the winter of 2015–2016 the southwest of England experienced a series of low-pressure weather systems. During the most significant, named storm Imogen (6–8 February 2016), maximum wind gust speeds of 127 km h^−1^ were measured (Culdrose, Cornwall (Met Office, 2016)) coinciding with 7.5 m maximum wave heights on the south coast of Cornwall (FaBtest, 2016). This storm caused significant damage to properties and infrastructure throughout the South West of the UK. During this storm the smaller A3 buoy and instrument string became disconnected from the main 100 L float. A local fisherman later recovered the A3 buoy and string and all scientific instruments were still attached and unharmed. After investigation it was found that the rope between the A3 buoy and the 100 L float had been cut, but it is unclear if this was due to the position of a split pin at the shackle with the A3 buoy (rubbing through the rope during the storm) or due to a boat becoming entangled in the mooring. To mitigate against the former situation, the mooring design was modified slightly to include an extra shackle to move the spliced rope away from the split pin.

Collectively, across the two deployments, the autonomous buoy system collected high quality data for 14 months in two near-shore coastal locations and these data are used below to discuss the suitability of the sites for i) mussel and lobster aquaculture and ii) allowing the growth of one type of biotoxin causing algae (*Dinophysis* spp.).

### 4.3 St Austell Bay

**Fig. 3:**
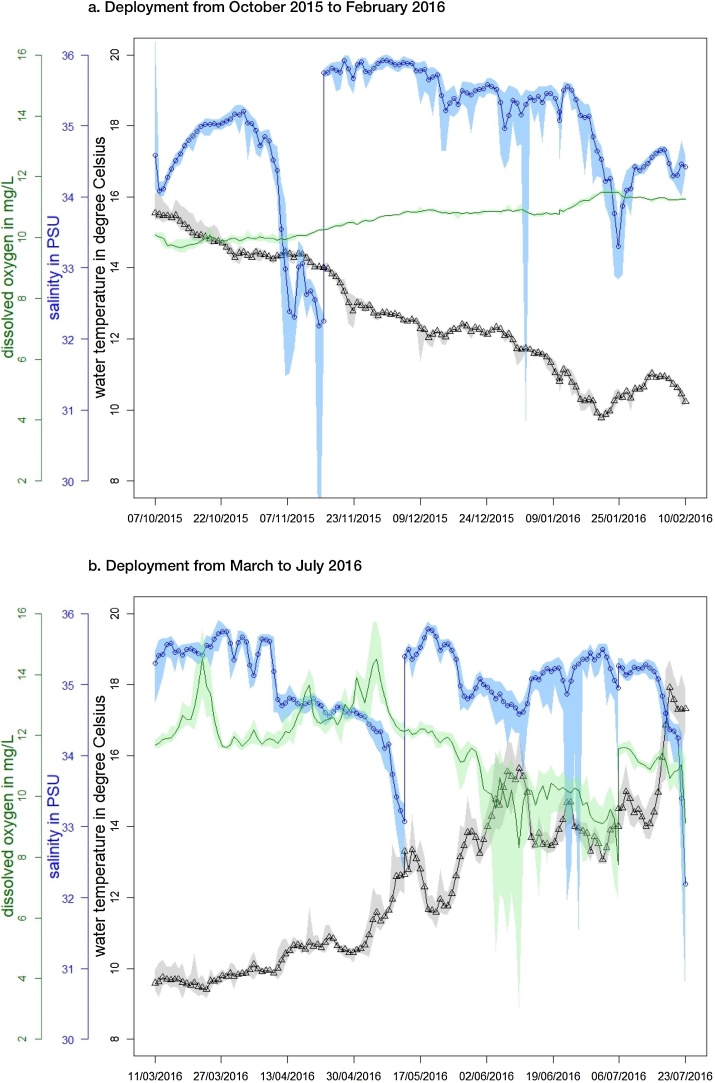
St Austell Bay daily mean sea temperature at 1.1 m (T in °C), mean salinity (in PSU) and mean dissolved oxygen (DO in mg L^−1^) from October 2015 to July 2016 (quality-controlled data). The mean sea temperature is shown as black line with triangles (grey shading represents the minimum and maximum sea temperature), the mean salinity is shown as blue line with circles (light blue shading represents the minimum and maximum salinity) and the mean DO is shown as green line (light green shading represents the minimum and maximum DO concentrations). (For interpretation of the references to colour in this figure legend, the reader is referred to the web version of this article.)

The data showed that within St. Austell Bay physico-chemical parameters change significantly on diurnal, weekly and seasonally scales (Fig. 3a and b), a characteristic that is unlikely to have been captured by a monthly or weekly sampling approach.

For example, diurnal changes in seawater temperature of about 1 ° Celsius were observed during October 2015 as well as during spring 2016 (March to April 2016). No thermal stratification between the three temperatures (located at different depths between 0.6 and 1.1 m) was observed, indicating that the top meter of the water column are well mixed throughout this period. This is in agreement with earlier observations by Sherwin et al. (1997), who described that thermal stratification within the water column in St. Austell bay occurs during calm wind conditions (less than 5 m s^−1^) and that otherwise the top 5–8 m are well mixed. The observed sea temperatures within this site are within the temperature range previously reported suitable for bivalve molluscs and lobster species (5–20 °C) (Gosling, 2003, Smith et al., 1999).

Salinity varied throughout the deployment time and ranged from 32.84 to 36.01 PSU. It is likely that these variations in the salinity can be attributed to the impacts of the River Par and other streams entering the bay that have been noted in the past to influence the near-shore dynamics of the bay (Sherwin et al., 1997). Within the bay tidal currents are very small (e.g. 0.024 m s^−1^) and it has been shown that there is a potential for a buoyant freshwater effluent (sporadic and assumed occasional) to be trapped at the surface of the water (Sherwin et al., 1997). Although bivalve molluscs, such as *Mytilus* spp., are well adapted to broad salinity conditions (5–32 PSU) (Gosling, 2003), it is also known that rapid change in the salinity of seawater can lead the mussels to close their shells and stop feeding (Berger and Kharazova, 1997). However, it is thought that the observed salinity range within at this site and during this deployment time would not adversely affect the growth of mussels, as observed changes in salinity (32.84–36.01 PSU) were short lived. Depending on oxygen conditions, the lower limit for adult lobsters ranges from 8 to 14 PSU (Dufort et al., 2001) and the observed salinity changes in St Austell bay were above this limit. Therefore, the salinity conditions are suitable for both bivalve molluscs and lobster aquaculture.

Dissolved oxygen is an indicator of eutrophication and an important metric for resident and transitory organisms. Through autumn and winter dissolved oyxgen concentrations ranged between 9.50–11.62 mg L^−1^, demonstrating oxygen saturation due to winter storm mixing of the water column. Shortly after redeployment of the buoy system in mid March as well as from mid April onwards dissolved oxygen concentrations reached high concentrations of 15 mg L^−1^, indicating the onset and development of the spring phytoplankton growth, ending around mid May when DO concentrations decreased to 12 mg L^−1^. It is known that if light and nutrients are sufficently present, then growth of phytoplankton in surface waters can supersaturate the water with DO (Best et al., 2007). Consequently, the decay of phytoplankton and the sinking of any material can result in oxygen depletion and, or reduction. On several occasions during June 2016 DO decreased below normal oxygen levels (6–10 mg L^−1^), with lowest observed concetrations of 2.99 mg L^−1^ (Fig. 3b). The lowest concentrations were observed during night, whereas observations during daylight showed higher oxygen levels (>6 mg L^−1^). This large variation in DO concentrations indicates the occurrence of a phytoplankton bloom during the June 2016 (O’Boyle et al., 2009, OSPAR, 2017). It has been shown that low levels of DO can affect marine organisms, for example, slowing growth rates and elevating stress levels in lobsters (Baden et al., 1990). Minimim oxygen requirements for lobster and other crustaceans have been shown to be between require a 1–4 mg L^−1^, whereas marine bivalves, such as mussels and oysters, require 1–2 mg L^−1^ oxygen (Best et al., 2007). The observed DO levels (Fig. 3) mean that the DO at this site is within the range needed for lobsters and bivalve molluscs to survive. Collectively, in terms of temperature, salinty and oxygen values and their temporal ranges, this site at water depths 0.6–1.65 m appears to be suitable for both bivale mollusc and lobster survival.

### 4.4 Fal estuary

**Fig. 4:**
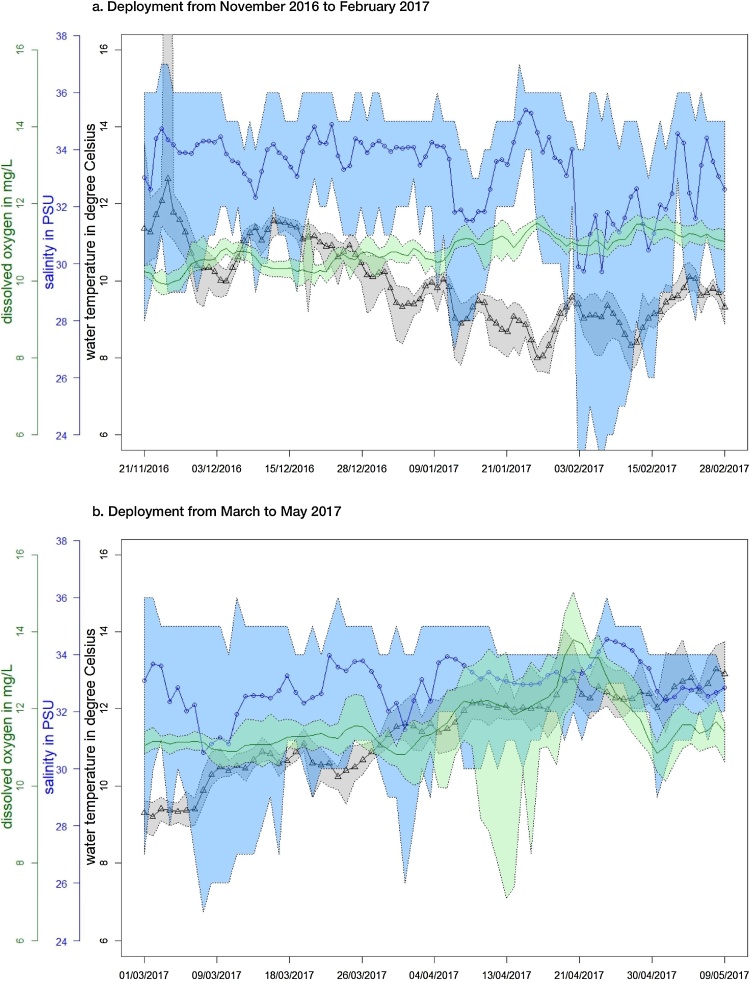
Fal estuary daily mean sea temperature at 1.1 m (T in °C), mean salinity (in PSU) and mean dissolved oxygen (DO in mg L^−1^) from November 2016 to May 2017 (quality-controlled data). The mean sea temperature is shown as black line with triangles (grey shading represents the minimum and maximum sea temperature), the mean salinity is shown as blue line with circles (light blue shading represents the minimum and maximum salinity) and the mean DO is shown as green line (light green shading represents the minimum and maximum DO concentrations). (For interpretation of the references to colour in this figure legend, the reader is referred to the web version of this article.)

The physico-chemical parameters for the Fal estuary are shown in Fig. 4a and b for the five-month deployment from November 2016 to May 2017. Sea temperature ranged from 7.6 to 14.1 °C, with lowest temperature recorded in January. As expected for this estuarine site, recorded salinity ranged broadly from 23 to 37 PSU and the variability is consistent with river flow and tidal influences. Over the five-month deployment DO concentrations were between 7–15 mg L^−1^ and, as for St Austell bay, oxygen increased and then reduced for the month April indicating phytoplankton growth. The recorded physico-chemical parameters described above suggest the suitability of this site for aquaculture, e.g. bivalve molluscs or lobsters.

### 4.5 Susceptibility of both sites for *Dinophysis* spp.

The phytoplnkton genus *Dinophysis* spp. is known to produce the biotoxin okadaic acid and its derivates, which can lead to closure of shellfish farms. Along with physico-chemical conditions that influence their survival, it is also hypothesised that increased abundance of *Dinophysis* spp. in the water column can be related to physical signals, including (diurnal) thermal stratification, halocline and pycnocline and the formation of frontal features (Farrell et al., 2012, Raine and McMahon, 1998, Reguera et al., 2012). Buoy observations during the St. Austell bay deployment showed that sea temperature increased by around 2 °C from 2nd to 8th July, indicating thermal stratification could have taken place within the farm (e.g. as the farm itself is likely to accelerate stratification by dampening vertical mixing). This period coincides with the observed occurrence of high concentrations of okadaic acid and its derivates, produced by *Dinophysis* spp. that was identified within the farm mussels by the routine agency sampling. Previous studies shown that *Dinophysis* spp. can thrive in waters with low salinity (i.e. <22 PSU) (Godhe et al., 2002), however it has been also reported that stratification of suffient magnitude and duration are important factors for *Dinophysis* spp. bloom initiation (Delmas et al., 1992). Water column straification has been previously observed within the Fal estuary (Sherwin, 1993) and thus could allow the growth of *Dinophysis* spp. at this site. Due to its tolerance to a wide range of sea temperature and salinity enables *Dinophysis* spp. to its wide geographically distrubtion (Reguera et al., 2012) and the recorded physico-chemical parameters of both study sites provide environmental conditions suitable for this genus.

## 5 Summary and conclusions

The novel low-cost, compact and robust autonomous buoy system has enabled the characterisation of the temporal variablity of physical, chemical and biological parameters of two contrasting near-shore coastal water. The design is able to survive gale force sea conditions. The characterised variations in the water quality parameters (temperature, salinity and DO), at both deployment sites confirm that (based on these parameters and depths) the two sites are suitable for the aquaculture of bivalve molluscs including mussels. In addition, both sites are also suitable for the cultivation of lobsters. The measurements have also confirmed that the physico-chemical conditions and the ability for stratification to occur in St Austell bay are also agreable for the existence of *Dinophysis* spp. (though only during the summer months). This is a toxin producing species, which when in the water can cause the accumulation of toxins within shellfish, leading to the short-term closure of the shellfish beds.

The novel buoy system could be used to characterise new aquaculture sites to evaluate their potential for farming and it could also be used within established farms to support farm management. For example, early warning of stratification conditions could guide the sale of farm stock, whilst the use of such monitoring methods will only work to increase customer confidence in the product.

The simple and generic design of the mooring makes it possible to add further instruments. To test this, a carbon dioxide sensor measuring partial pressure of carbon dioxide was added for a two-month period, which in conjunction with the salinity and temperature sensors allowed the coastal carbonate system to be investigated; these results will be reported elsewhere. Such capability is likely to become increasingly important for shellfish aquaculture, as sudden changes in the carbonate system (e.g. due to the upwelling of cold water rich in carbon dioxide) have been shown be detrimental to mussel shell growth (Fitzer et al., 2014) and oyster spat production (Barton et al., 2012).

The generic design, simple operation, and low cost approach lends itself to being used by non-scientific operational agencies responsible for monitoring coastal bathing waters. For example, the autonomous buoy system including durable and accurate sensors could be used towards cost-effective confirmation of conditions conducive to high algal concentrations in the near-shore waters and subsequent confirmation of the die off of any resulting algal bloom.

## Authors Contributions

The buoy system was designed and built by W.S., D.R., D.P., I.G.C.A. and J.D.S.; W.S. analysed the data, W.S. and J.S. drafted the manuscript and all authors contributed; C.J.A.C. and P.I.M provided guidance on the buoy’s application to ShellEye.

## Conflict of interest

The authors declare no conflict of interest.
